# In/Out Status Monitoring in Mobile Asset Tracking with Wireless Sensor Networks

**DOI:** 10.3390/s100402709

**Published:** 2010-03-26

**Authors:** Kwangsoo Kim, Chin-Wan Chung

**Affiliations:** 1 RFID/USN Research Department, Electronics and Telecommunications Research Institute (ETRI), 138 Gajeongno Yuseong-gu, Daejeon, 305-700, Korea; E-Mail: enoch@etri.re.kr; 2 Department of Computer Science, Korea Advanced Institute of Science and Technology (KAIST), 335 Gwahakro, Yuseong-gu, Daejeon, 305-701, Korea

**Keywords:** wireless sensor networks, mobile asset tracking, mobile node, status monitoring, node failure detection

## Abstract

A mobile asset with a sensor node in a mobile asset tracking system moves around a monitoring area, leaves it, and then returns to the region repeatedly. The system monitors the in/out status of the mobile asset. Due to the continuous movement of the mobile asset, the system may generate an error for the in/out status of the mobile asset. When the mobile asset is inside the region, the system might determine that it is outside, or *vice versa*. In this paper, we propose a method to detect and correct the incorrect in/out status of the mobile asset. To solve this problem, our approach uses data about the connection state transition and the battery lifetime of the mobile node attached to the mobile asset. The connection state transition is used to classify the mobile node as normal or abnormal. The battery lifetime is used to predict a valid working period for the mobile node. We evaluate our method using real data generated by a medical asset tracking system. The experimental results show that our method, by using the estimated battery life time or by using the invalid connection state, can detect and correct most cases of incorrect in/out statuses generated by the conventional approach.

## 1. Introduction

The development of information technology has created a demand for small sized computing devices. These are placed at specific positions, capture data around them, communicate with neighbors, and transmit the data to others via wireless communications [1]. They are called sensor nodes and collectively they form WSNs (Wireless Sensor Networks). Each node becomes a sensing device as well as a routing device in sensor networks.

Many valuable applications using WSNs have been implemented in recent years. These applications are categorized into two groups, according to the type of sensor node that the applications use. There are stationary sensor networks and mobile sensor networks. Stationary sensor networks consist of a number of static nodes. In such a network, the sensor nodes are placed at different locations to monitor environmental conditions; the positions of the sensor nodes rarely change. Such systems are applied to structural health monitoring [2,3], environmental monitoring [4,5] building management [6], static asset management [7], traffic monitoring [8], and factory automation [9]. Mobile sensor networks, on the other hand, contain one or more mobile sensor nodes. In such a network, each mobile node is attached to a mobile object. The movement and the current position of the object are monitored in real time. This system enables users to find and count mobile objects quickly, and it can be applied to mobile asset tracking [10,11], human monitoring [12], and medical equipment monitoring [13].

The mobile asset tracking system monitors movements and locations of mobile assets within a monitoring area by attaching a mobile node to a mobile asset. The position of the mobile node is considered as that of the mobile asset. The mobile asset can move around within the monitoring area, stay in another place after leaving that area, and then return to the original area. The continuous movement of the mobile asset changes the configurations of WSNs by changing its parent node, causes the asset to disconnect from the network, and makes the system generate incorrect results. One of these changes of WSNs is a possible error in the in/out status of the mobile asset. The system might determine that the position of the mobile asset is outside the area even when the asset is within the area, or vice versa. The incorrect in/out status of the mobile asset is caused by node failure. However, the above works that use mobile sensor networks do not describe the effect of node failure while the applications are working; rather, they assume that all nodes are reliable. For example, the mobile asset tracking system described in [10] is used to monitor mobile medical equipment in the emergency room of Incheon Gil Medical Center in Korea. The emergency room has a lot of medical equipment necessary to provide medical treatment to patients with various illnesses and injuries according to the patient’s condition. In real time, the system monitors several mobile medical assets with mobile nodes and reports their positions to nurses. The system allows the nurses to rapidly generate a report that details how many assets are in the emergency room and how many assets have left the area, as well as where the mobile assets are. However, a few nurses have reported that the number of inside and outside assets determined by the system were different in real situations while the system was working.

There has been some research on controlling false status, as will be discussed in Section 2. These projects have tried to detect node failures using observer nodes that monitor whether target nodes perform their functions or not. The methods are suitable for static nodes because the relationship between observer nodes and target nodes is constant and they exchange monitoring messages periodically. However, such a method cannot be applied to mobile nodes because the relationship between the observer and the target nodes changes continuously when mobile nodes move.

In this paper, we investigate the reason for the generation of false reports in the mobile asset tracking system described in [10] and determine that they are generated by incorrect in/out status reports of the mobile nodes attached to the mobile medical assets. This paper describes how to recognize and correct the incorrect in/out status of a mobile node on an application server that is located outside the WSN. We detect two failures that cause incorrect in/out status of the mobile node: abnormal connection state and exhausted battery. We use connection state and battery lifetime of the mobile node to detect the failures. When the mobile node moves, its connection state changes and can be classified into a state of joined, left, or transitive. We detect the abnormal connection state of the mobile node if it stays in the transitive state for more than a certain amount of time. Each mobile node has a battery that has a limited lifetime. Mobile nodes consume different amounts of energy according to their in/out statuses. We estimate the energy consumption of the battery and detect abnormal nodes from the exhausted battery. Detecting the incorrect in/out status is very important in the mobile asset tracking system. False in/out status determination turns users away from the system because they feel they cannot trust its reliability. Incorrect status should be identified and corrected to increase system reliability as well as customer satisfaction. In summary, the contributions of this paper are as follows:

♦ We develop a framework to detect and correct the incorrect in/out status of a mobile node in a mobile asset tracking system based on the properties of mobile nodes. This method is applicable to many mobile asset tracking applications. To our knowledge, this is the first incorrect in/out status detection technique for mobile asset tracking systems.
♦ We propose two state classifiers to control the incorrect in status of a mobile node. These are network based and frequency based classifiers that categorize mobile nodes as either a normal node with a normal connection state or an abnormal node with an abnormal connection state.
♦ We propose a battery lifetime estimator to control the incorrect out status of a mobile node. This method estimates battery lifetime and categorizes nodes as either a normal node with a good battery level or an abnormal node with an exhausted battery.
♦ We perform experiments with real data sets using both state classifiers and battery lifetime estimation. The experiments show that our approach not only detects incorrect in/out status but also corrects it accurately.

The rest of the paper is organized as follows. Section 2 presents the related work and background. Section 3 presents the system architecture of the mobile asset tracking system and describes its components. Section 4 explains our methods for tackling the problem. Section 5 describes our experimental environment and compares the proposed method with an existing method. Finally, Section 6 concludes the paper.

## 2. Related Work

This paper studies the incorrect in/out statuses of mobile assets caused by unexpected node failures. Most previous works focus on detecting static node failures. This paper is to look at detecting mobile node failures and linking those failures to the incorrect in/out statuses of mobile assets for mobile asset tracking systems. In this section, we will briefly review some work on node failure detection methods in sensor networks.

Chen *et al.* [14] propose a distributed fault detection method that determines the status of a node by testing the difference of the sensed data produced mutually by neighbor nodes. When two neighbor nodes receive sensed data transmitted by each node, each node calculates the difference between the received data and its own sensed data. If the difference exceeds a certain threshold value, one node determines that the other node is a failure node. However, this method cannot be applied to mobile nodes. Since mobile nodes move continuously, the mobile nodes cannot be used to determine individual node failure even when the difference of data among neighbor nodes exceeds the threshold.

Ramanathan *et al.* [15] propose Sympathy, a tool for detecting and debugging failures in sensor networks. Sympathy runs at each node, which transmits monitored traffic and metrics traffic. The former is produced by the sensor node in the sensor network and the latter is produced by Sympathy. Sympathy monitors the traffic to detect failure nodes and determine the source of the failure. When a node generates less monitored traffic than a certain threshold, Sympathy determines that the node is a failure node. The failure detection occurs at a sink node. If this method is applied to mobile nodes, Sympathy would classify all mobile nodes that leave a sensor network as failure nodes because the sink cannot receive any messages from them. All mobile nodes would produce incorrect determinations, and so Sympathy is not applicable to mobile nodes.

Rost and Balakrishnan [16] and Meier *et al.* [17] propose Memento and DiMo, respectively. Memento and DiMo are network management systems for wireless sensor networks. These methods separate all nodes into two groups: observer nodes and target nodes. Each node periodically sends heartbeat messages to its neighbor nodes. The observer node monitors whether the target node is alive or not. If the observer does not receive a heartbeat message within a certain monitoring time from the target node, then the observer node considers the target node to have failed. These researchers also propose methods that consider wireless packet losses to reduce the false positive rate. In DiMo, the target node sends a recovery message to the sink node via another node when the target node is alive but cannot send any messages to the observer node. Memento develops a variance-bound based failure detector that uses the mean and standard deviation of the number of missing heartbeats. Memento and DiMo do not consider the idea that mobile nodes can leave a sensor network. When an observer node monitors a mobile node, the mobile node can leave the monitoring area. Then, the observation relationship is broken and the observer node considers the mobile node as a failure node, even though the mobile node is alive.

Since they do not consider the movements of mobile nodes, the above in-network approaches cannot be applied to monitor mobile nodes. Therefore, we have to devise a new node failure detection method for application monitoring mobile nodes. As a mobile node can move within a monitoring area, stay in another place after leaving that area, and then return to the original area, the new method should not classify a mobile node that leaves a sensor network as a failure node. The new method runs on a centralized server, located outside the sensor network, in order to detect mobile node failures correctly.

## 3. System Architecture

**Figure 1.**
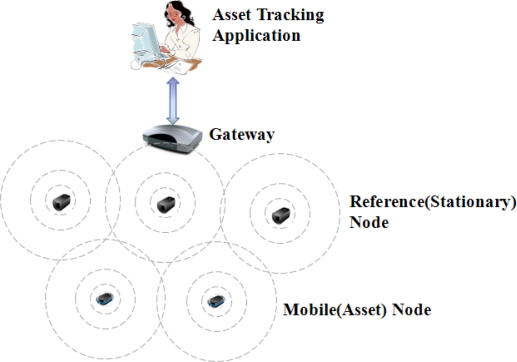
System Architecture.

We describe the system architecture and features of each component. The architecture is shown in Figure 1 [10]. The system consists of 4-tuple S = (A, G, R, M) where A is an application; G is a gateway; R = {r_1_, r_2_,..., r_m_} is the finite set of reference nodes; and M = {m_1_, m_2_,..., m_n_} is the finite set of mobile nodes.

The mobile nodes are located at the lowest level and attached to mobile assets such as medical equipment, hot coils, ship blocks, staff members, and visitors. The mobile node is a battery powered device and has a battery conserving sleep mode. The location of the mobile node is considered to be the position of the mobile asset. The mobile node receives RSSIs (Radio Signal Strength Indicators) from the reference nodes and sends them to the gateway periodically. The RSSIs will be used by the gateway to calculate the position of the mobile node. Initially, each mobile node becomes a child node of the one reference node that sends the strongest RSSI. The mobile node selects another parent node when it cannot receive any signal from the previous parent.

The reference nodes are stationary nodes and form the infrastructure of the wireless sensor network. They are deployed in a monitoring area, provide high spatial resolution, and monitor mobile nodes. Each reference node knows its own location and exchanges messages with its neighbor nodes. Reference nodes play roles of route nodes routing messages. When reference nodes receive messages from mobile nodes, they relay those messages to the gateway, and also relay control messages received from the gateway to the mobile nodes.

The gateway becomes a bridge between the wireless sensor network and the application through an IP-based network. The gateway receives the messages related to the locations of the mobile nodes from the reference nodes and calculates their locations. The calculated locations are transmitted to the application. The location data contains data of x, y, and timestamp. The x and y are the position of a mobile node and the timestamp is the time at which the gateway calculates the position. The gateway sends a connection state of the mobile node to the application when the connection state of the mobile node changes.

The asset tracking application is in the highest level of the architecture. The entire monitoring area is given to the application, which connects to the gateway and acquires the locations and the connection states of all mobile nodes. This system keeps track of the current positions of the nodes and determines whether the nodes are inside or outside the monitoring area. The application displays their current positions and in/out statuses on the user interface. Therefore, the application is used for two services: asset finding and asset management. The asset finding service requires the approximate position of each mobile asset and the asset management requires the exact number of inside and outside mobile assets. This paper describes issues related to the asset management.

## 4. Status Failure Detection

### 4.1. Problem Definition

In asset management, an application determines the status of each mobile asset using a WITHIN function. We will describe the basic concept.

Let R be the boundary of a monitoring region. There are several mobile assets to be monitored and their locations are loc_1_, loc_2_,..., loc_i_. Then, the status, s_i_, of each mobile asset can be determined as inside or outside the space by using a WITHIN(R, loc_i_) function written as Equation (1):

(1) $$s_{i}=\left\{ \begin{array}{l} \mathrm{Inside}\;\;\;\;\;\mathrm{if}\;\mathrm{WITHIN}\left(R,{\mathrm{loc}}_{i}\right)==\mathrm{true}\\ \mathrm{Outside}\;\mathrm{otherwise} \end{array}\right.$$

The WITHIN(R, loc_i_) function in Equation (1) tests whether the loc_i_ is inside R or not. If a mobile node does not report its position, then the application determines that it is outside R. In many applications utilizing WSNs, there may be difficult environmental conditions such as communication barriers, physical obstructions, and electromagnetic noise, any of which may cause a node to fail or temporarily disconnect from a sensor network. Therefore, the determined positions or in/out statuses of the mobile assets in the mobile asset tracking application might be incorrect. However, most applications utilizing WSNs assume that all nodes are stable. The incorrect in/out status is summarized in Table 1. The state transition error and the exhausted battery will be handled in the paper.

### 4.2. Connection State Model

**Figure 2.**
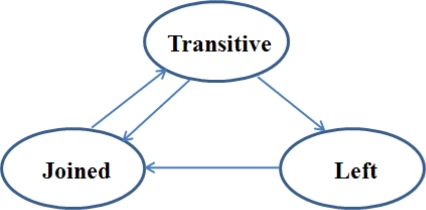
State transition diagram.

Each mobile asset with a mobile sensor node can move here and there within a monitoring area, stay in another place after leaving that area, and then return to the original area. When a mobile asset changes its position, the connection state of the mobile node changes. This change is reported by the gateway. The connection state (CS) is the set of possible states that the mobile node can have and is defined as *CS = {Joined, Transitive, Left}*. The mobile node has only one of the connection states at a specific time. This situation is shown in Figure 2.

A mobile node in the joined state connects to a sensor network and exchanges messages with its parent node. The location of the mobile node will change when it moves. Every node starts from the joined state because the sensor network can recognize the node as one of its members after it connects to the network.

A mobile node in the left state disconnects from the sensor network. Therefore, it cannot exchange any messages with other nodes. It is trying to rejoin the network. When it finds a connection to a parent node, its state is changed from left to joined.

A mobile node in the transitive state has disconnected from the sensor network temporarily due to a movement from its current position to another place or that it has encountered unstable communication. If the mobile node moves out of the radio range of its parent node, it has to change its parent node. Therefore, it cannot send any message to the gateway until it finds a new connection to the network. The location of the node will not change and the gateway reports the previous position of the node because the gateway cannot determine its current location. Since it has been trying to rejoin the network, the state of the node will eventually become either joined or left. The condition of the state transition from the transitive state is described as Equation (2):

(2) $$\mathrm{CS}=\left\{ \begin{array}{l} \mathrm{Joined}\;\;\;\;\;T_{\mathrm{tr}}\leq T\;\mathrm{and}\;\mathrm{if}\;a\;\mathrm{node}\;\mathrm{rejoins}\;\mathrm{the}\;\mathrm{network}\\ \mathrm{Left}\;\;\;\;\;\;\;\;T_{\mathrm{tr}}>T\;\mathrm{and}\;\mathrm{if}\;a\;\mathrm{node}\;\mathrm{can't}\;\;\mathrm{connect}\;\mathrm{to}\;\mathrm{the}\;\mathrm{network}\; \end{array}\right.$$

where T_tr_ is sojourn time of a mobile node in the transitive state and T is the threshold.

**Figure 3.**
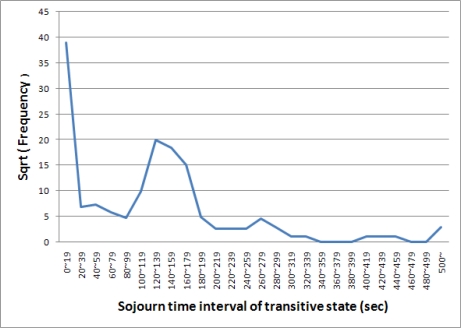
Example of distribution map of sojourn time intervals in the transitive state.

A mobile node should change its state from transitive to joined before time T or to left after time T. We produced a distribution map, shown in Figure 3, to understand the characteristic of the sojourn time in the transitive state of nodes. Figure 3 shows the sojourn time interval and the frequency with which nodes stay in the transitive state during the period. Data were received from all asset nodes for ten days. The x-axis presents the sojourn time interval of the transitive state and the y-axis displays the frequency of each interval. For example, nodes sojourn in the transitive state about 400 times between 120 and 139 seconds.

### 4.3. State Classifier

In this section, we will explain how to solve the first case, in which an asset exists outside the monitoring area but the application determines that it exists inside. We will introduce two methods that use the connection state to rectify this problem.

From the distribution map, we can recognize that some nodes in the transitive state keep their states for a long time without any state transition. The long transitive state is caused by a gateway. If the gateway misses a node’s connection state message, the gateway sends the previous connection state of the node continuously until a new connection state message arrives from that node. This method has an advantage in that it prevents a user from recognizing the temporal disappearance of the node. If the gateway misses the connection state message continuously, the method generates an error, telling the user that the node has stayed in the same position even though the node has moved to another place. Therefore, we consider a node with a long transitive state as an abnormal node. The detected abnormal node will be reported to the user and the network administrator. We have to determine a threshold that separates the normal and the abnormal states. This threshold is called the STO (sojourn timeout) in this paper. We propose two state classifiers to determine the threshold value.

#### 4.3.1. Network-Based Classifier (NBC)

Our initial approach to the design of the state classifier uses attributes of a sensor network. We call this classifier a network-based classifier. When a mobile node moves from one place to another and escapes from the radio range of its parent node, the parent node detects that it cannot exchange any message with the node. Then, the parent node waits for a certain amount of time to determine whether the child has disconnected from the system or not. The parent node reports a node drop message to a gateway if it cannot receive any message from the child after a certain amount of time has expired. When the gateway receives the node drop message, it changes the connection state of the dropped node from joined to transitive. Then, the gateway waits a given time to decide whether the dropped node has disconnected from the sensor network. If another reference node reports that the dropped node has connected to it, then its connection state will be changed from transitive to joined. If the given time expires and the gateway does not receive a join message from any reference nodes, then the gateway changes the original node’s connection state from transitive to left and reports this new connection state to the application.

A valid sojourn timeout in the transitive state of a mobile node can be calculated according to Equation (3):

(3) $$T_{l}\leq \text{STO}\leq T_{u}$$

$$T_{l}=T_{a}+T_{e}+d\times T_{p}+d\times T_{m}$$

$$T_{u}=T_{a}+T_{e}+T_{n}+d\times T_{p}+d\times T_{m}$$

♦ T_a_ : local waiting time between a parent node and a mobile node
♦ T_e_ : network-wide waiting time at the gateway
♦ d : depth of tree
♦ T_p_ : propagation delay time between nodes
♦ T_m_ : message processing time at a node
♦ T_n_ : connection construction time required to set up a connection anew when a node joins the network

One value is selected from [T_l_, T_u_] as STO. The STO is used to detect abnormal nodes. If a node’s real sojourn time in the transitive state is longer than STO, it is considered an abnormal node with an abnormal state. As the separation of the normal and the abnormal states starts before the reports on the connection states of mobile nodes is complete, if STO is less than T_l_, the short STO might generate an incorrect determination about the normal or abnormal states of mobile nodes. Therefore, some normal nodes will be considered abnormal nodes. If STO is larger than T_u_, the long STO might also generate an incorrect determination because the separation of the normal and the abnormal states is delayed. Therefore, abnormal nodes are considered as normal nodes until the separation process finishes.

#### 4.3.2. Frequency-Based Classifier (FBC)

The network-based classifier uses a fixed sojourn timeout when the nodes and the gateway are initialized. Therefore, it might not adopt the characteristics of movements of the mobile nodes in the sensor network. If the sensor network monitors a wide area, the sojourn time in the transitive state of a mobile node moving from one side of the network to another might be longer than the fixed sojourn timeout that is used by NBC. This might cause unexpected results. We propose another classifier that adopts the real movements of mobile nodes within the sensor network. We call this classifier a frequency-based classifier; it uses frequency data extracted from accumulated movement data.

We will consider a sojourn time with a low frequency as an abnormal state, as this situation rarely occurs. However, it is difficult to extract a threshold to separate the normal state and the abnormal state from the frequency data because sojourn times with high frequencies, low frequencies, and zero frequencies are mixed in the data without any rule. Therefore, the classifier transforms the frequency data by increasing high frequencies and attenuating low frequencies to separate the sojourn times into normal state and abnormal state. It will be easy to select a threshold from the transformed frequency data because the low frequencies will be zero after the transformation.

Consider a set *S = {(t_1_, f_1_), (t_2_, f_2_),..., (t_n_, f_n_)}*, where *t_1_ < t_2_ < ... < t_n_*, t_i_ indicates the sojourn time that a node stays in the transitive state, and f_i_ indicates the frequency that represents the number of nodes staying in the transitive state during the sojourn time. This information is collected from the sensor network. We transform S into S′ with the frequency-based classifier to categorize the sojourn times into the normal state and the abnormal state. Then, we get the transformed set *S′ = {(t_1_,f′_1_), (t_2_,f′_2_),..., (t_n_,f′_n_)}*, where f′_i_ indicates a transformed frequency for the sojourn time. We separate S′ into two sub sets, S′_1_ and S′_2_ : *S′_1_ = {(t_1_,f′_1_), (t_2_,f′_2_),..., (t_i_,f′_i_)} with ∀(t_j_,f′_j_) ∈ S′_1_,f′_j_ > 0, S′_2_ = {(t_i+1_, f′_i+1_), (t_i+2_, f′_i+2_),..., (t_n_,f′_n_) with ∀(t_k_,f′_k_) ∈ S′_2_,f′_k_ = 0*, where t_1_ < t_2_ <...< t_i_ < t_i+1_ <...< t_n_.

**Figure 4.**
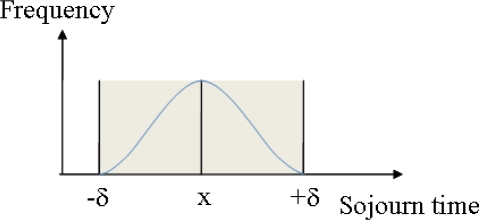
Graphical illustration of the scoring function.

The frequency based classifier consists of two functions: scoring function and density function, which transform the collected frequencies into the new frequencies. The scoring function describes the effect of the frequencies within its neighborhood on the data point. It is written as Equation (4):

(4) $$f\left(x,y\right)=\{\begin{array}{ll} e^{-\frac{{\left(x_{f}-y_{f}\right)}^{2}}{2\times \delta^{2}}} & \mathrm{if}\;\left|x-y\right|\leq \delta \\ 0 & \mathrm{otherwise} \end{array}$$

where x_f_ and y_f_ are frequencies of sojourn times x and y in the transitive state, respectively, δ controls the width of neighborhood, and y ∈ [x − δ, x + δ]. Figure 4 shows the scoring function graphically.

An important factor is that the data that falls within [x − δ, x + δ] actually affects the density and all other data are neglected. The density function at the sojourn time x is defined as the sum of the scoring functions of all neighbors within [x − δ, x + δ]. The function is written as Equation (5):

(5) $$f\left(x\right)=\sum_{i=1}^{N}f\left(x,x_{\;f}^{i}\right)=\sum_{i=1}^{N}e^{-\frac{{\left(x_{f}-x_{\;f}^{i}\right)}^{2}}{2\times \delta^{2}}}$$

where x^1^_f_ is the frequency of the sojourn time x − δ and x^N^_f_ is that of the sojourn time x + δ.

If δ increases, more and more data affects the frequency of x. On the other hand, the density of x might be equal to zero if only x exists within [−δ, +δ] and there is no other data within the boundary. Therefore, the density of the sojourn time, which rarely happens, is very low or equal to zero. The classifier separates the sojourn times into two consecutive groups using the density values: normal state and abnormal state. The normal state has a certain frequency value and the abnormal state has a frequency value equal to zero.

**Lemma.** Let X be the sojourn time in the transitive state of a node, and let t be a sojourn timeout larger than 1. Then, the probability that the node stays in the transitive state longer than t is less than or equal to $\frac{E(X)}{t}$. The proof follows by applying Markov's inequality to obtain the probability, as shown in Equation (6):

(6) $$P\left(\left|X\right|\geq t\right)\leq \frac{E\left(X\right)}{t}$$

**Figure 5.**
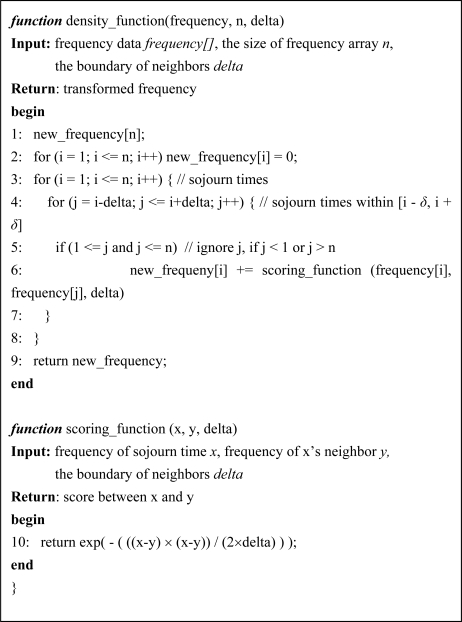
Density and scoring functions.

**Figure 6.**
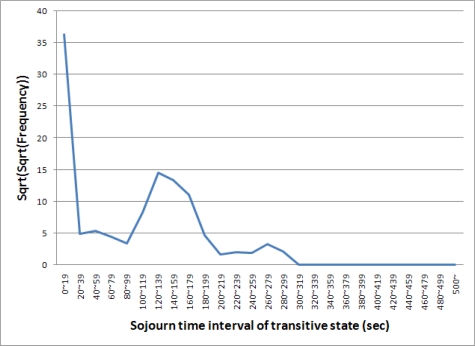
An example of the frequency transformation with δ = 3.

The procedures of the scoring function and the density function are described in Figure 5. In Figure 5, the density function selects frequencies of a sojourn time x and its neighbors within [x − δ, x + δ] and calculates the transferred frequency of x from line 4 to line 7. The scoring function then calculates the score between x and its neighbor at line 10. The frequencies in Figure 3 are transformed by the two functions and the result is shown in Figure 6. In Figure 6, we can separate the sojourn time intervals into two groups: from 0 to 299 and from 300 to 500. The sojourn time intervals of the former have frequencies larger than zero and those of the latter have frequencies equal to zero. We consider the former as the normal state and the latter as the abnormal state.

### 4.4. Battery Lifetime

In this section, we will explain how to solve the second case, in which an asset is inside the monitoring area but the application determines that it is outside. We will introduce a method that uses the battery lifetime.

Each mobile node is a battery powered device. The battery has a limited lifetime. If the battery is exhausted, the mobile node cannot join the sensor network. Therefore, the application can determine that the asset is outside the area even though it is actually inside, because the application cannot receive the location of the mobile node from the gateway. To overcome this unexpected result, we propose a battery lifetime estimator that predicts the battery lifetime of the mobile node and uses the estimated lifetime to determine the correct status of the mobile node.

**Figure 7.**
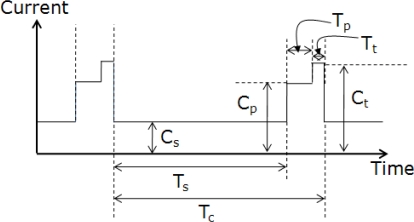
Approximation of energy consumption phases.

The amount of energy consumption of sensor nodes is different according to the status of the nodes. The approximated energy consumption phases are given in Figure 7 and the variables are listed in Table 2.

The energy consumption of the inside node can be divided into three phases: sleeping, polling, and transmitting. The node spends most of its time in the sleeping phase, during which the microcontroller and radio are utilizing low power states and the device is waiting for a timer to expire to wake it. When the device wakes, it enters the polling phase, during which the node exchanges beacons with its parent node to notify that it is alive and collects RSSIs from reference nodes. Finally, in the transmitting phase, the node sends signals to its parent node. After sending the data, it goes into the sleeping phase. The procedure is repeated periodically. The transmitting and polling phases consume an order of magnitude more energy than the sleeping phase and the transmitting phase expends the largest amount of energy.

The energy consumption of the outside node can be divided into two phases: sleeping and polling. The sleeping and polling phases are repeated periodically. The transmitting phase does not happen because the node cannot find any connection to the sensor network.

The amount of energy consumption of the inside and the outside nodes is different. The inside node consumes more energy than the outside node. The sleeping time of the outside node is shorter than that of the inside node, in order to allow it to rejoin the network quickly. The polling time of the outside node is also shorter than that of the inside node, in order to reduce energy consumption. Transmitting does not occur to reduce energy consumption when the node is outside the monitoring area.

We will estimate the consumed current and the predicted battery life times of an inside node and an outside node as follows.

The average consumed current, C_avg_, during unit time of an inside node is written as Equation (7):

(7) $$C_{\mathrm{avg}}=\frac{\left(T_{p}\times C_{p}\right)+\left(T_{t}\times C_{t}\right)+\left(T_{s}\times C_{s}\right)}{T_{c}}$$

The predicted battery life time, T_b_, of the inside node is defined as Equation (8):

(8) $$T_{b}=\frac{C_{b}}{C_{\mathrm{avg}}}$$

The average consumed current, C′_avg_, during unit time of an outside node is calculated as Equation (9):

(9) $${C^{\prime }}_{\mathrm{avg}}=\frac{\left({T^{\prime }}_{p}\times {C^{\prime }}_{p}\right)+\left({T^{\prime }}_{s}\times {C^{\prime }}_{s}\right)}{{T^{\prime }}_{c}}$$

The predicted battery life time, T′_b_, of the outside node is defined as Equation (10):

(10) $$T\prime_{b}=\frac{C_{b}}{C\prime_{\mathrm{avg}}}$$

The remaining current, C_r_, of a mobile node at the present time is estimated as Equation (11):

(11) $$C_{r}=C_{b}-\left(\left(C_{\mathrm{avg}}\times \sum_{i=1}^{N}T_{i}\right)+\left(C\prime_{\mathrm{avg}}\times \sum_{j=1}^{M}T_{j}\right)\right)$$

where T_i_ is the duration that the node stayed inside the monitoring area and T_j_ is the duration that the node stayed outside.

The estimated life time, T_e_, of the mobile node is calculated as Equation (12):

(12) $$T_{e}=\frac{a\times C_{r}}{C_{\mathrm{avg}}}+\frac{b\times C_{r}}{C\prime_{\mathrm{avg}}},\text{where}\;\;\;\;a=\frac{\sum_{i=1}^{N}T_{i}}{\sum_{i=1}^{N}T_{i}+\sum_{j=1}^{M}T_{j}},\;\;\;b=\frac{\sum_{j=1}^{M}T_{j}}{\sum_{i=1}^{N}T_{i}+\sum_{j=1}^{M}T_{j}}$$

We can detect an exhausted battery by using the estimated life time T_e_ and the remaining current C_r_. We decide that a node has an exhausted battery if T_e_ is less than the specified life time. This means that the battery will die before the end of the specified life time. If the remaining current is less than the specified remaining current, the battery cannot guarantee the normal operation of the node even if the current remains. The specified remaining current is calculated as follows: b_e_ × C_b_, where b_e_ represents the efficiency rate of the battery that is inserted into the node. Therefore, we decide that a node has an exhausted battery if C_r_ < b_e_ × C_b_. We will inform the network administrator and the user that the node’s battery is exhausted.

## 5. Performance Evaluation

In this section we discuss our experimental environment and results based on real data.

### 5.1. Experimental Environment

**Figure 8.**
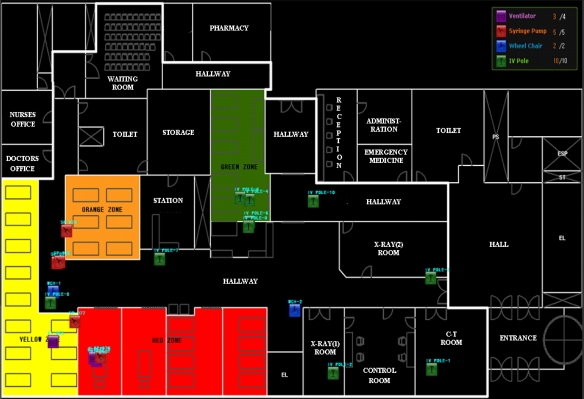
Map of the emergency room.

To show the practical significance of our new approach, we performed experimental evaluations of the approaches. They were executed on a trace of real data generated by a mobile asset tracking system. The system had been operating in the emergency room of the Incheon Gil Medical Center in Korea to monitor mobile medical equipment. It was designed to satisfy several requirements given by nurses who work in the emergency room. The map of the emergency room is shown in Figure 8. It includes four sections that are partitioned according to patient types treated in them, two X-Ray rooms, a CT (Computed Tomography) room, a control room for X-Ray and CT operations, a staff station, nurses’ and doctors’ offices, a waiting room, a hallway, and a storage. We used four different types of mobile medical assets: ten IV poles, four ventilators, five syringe pumps, and two wheelchairs. Each mobile node was attached to each mobile asset. The component and legend of the system are summarized in Table 3.

The system was installed in the emergency room 50 m × 40 m, which is partitioned by concrete walls. As the walls affect the signal propagation among the nodes, we carried out two tasks: simulation and measurement. We made a deployment plan for the reference nodes by a simulator. And we put the nodes on their positions selected by the deployment plan and measured the actual signal strength from the reference nodes at different positions within the emergency room. If the signal strength from reference nodes was very different from an expected value, then some of the reference nodes were moved to other positions. The nodes’ moved positions using the measurement were somewhat different from the positions using the simulation because the simulation did not consider the walls. Therefore, the final positions of the reference nodes were selected by the actual measurement.

The system had been maintained every two weeks and exhausted batteries were changed during the maintenance period. The maximum number of nodes that the gateway can have is 65,535. The maximum number of children of each reference node is eight. On the average, the accuracy of the location algorithm using RSSI is 2.5 m. The accuracy is enough to satisfy the nurses because an asset with the error boundary can be recognized by the nurses. However, the location algorithm is not discussed in this paper because it is not our research issue. The beacon exchange and the location update occur together at each mobile node to reduce energy consumption. Each mobile node wakes up every 30 seconds and exchanges beacons with its parent to confirm that the connection between the two nodes is valid. After confirming the connection, the node collects RSSIs from its neighbor reference nodes and transmits them to its parent. After sending the data, the node goes into the sleeping phase.

The nurses had been satisfied with the location accuracy of assets but sometimes complained about the incorrect statuses of them. They counted the number of inside and outside assets twice: by themselves and through the system, and produced some reports containing the number and the name of assets with the incorrect status.

We acquired the nurse’s reports and the log data that the system had generated from May 2007 to September 2007. The system determined the statuses of mobile nodes by using node positions during the period. Ten days amount of data from 1 July to 10 July are used to analyze the cause of incorrect status. All dates on which the incorrect status events occurred are extracted from the nurses’ reports and also both the connection state and the location data of each mobile node for the dates are extracted from the log. We apply our method to the log data of the dates on which the incorrect status occurred and compare the in/out status in the log data to that produced by our method. The nurses’ reports are used to confirm the correctness of our method.

### 5.2. Status Change from Inside to Outside

We evaluate the impact of transitive state by varying the sojourn timeout. STO is used to separate the normal node and the abnormal node. If the sojourn time in the transitive state of a mobile node is larger than STO, the state classifiers determine that the node is an abnormal node and change its connection state from transitive to left. State classifiers also change its status from inside to outside. We use two methods, the network-based classifier and the frequency-based classifier, to determine the STO value.

**Figure 9.**
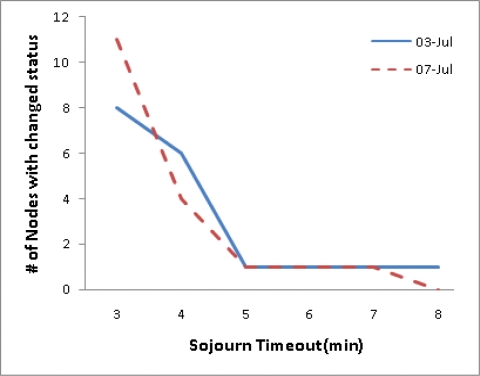
Number of nodes with changed status from inside to outside for different STOs.

**Impact of the sojourn timeout**: We vary STO between three and eight minutes in increments of one minute. Figure 9 shows the number of nodes that change status from inside to outside according to varying STO. For example, if the STO is set to three minutes, the status of eight nodes on 3 July will be changed from inside to outside. Decreasing STO increases the number of nodes whose status is changed from inside to outside. The number of nodes with changed status is rapidly changed before five minutes; however, it is nearly changeless after the time. From this fact, we can infer that most of state transitions occur before five minutes. Therefore, the state transition after five minutes might be an abnormal state.

**Figure 10.**
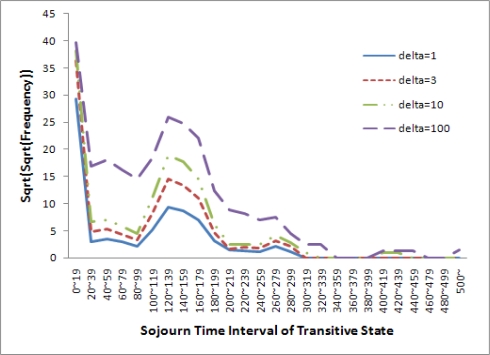
Example of frequency graph for different δ.

**Impact of δ**: We evaluate the impact of δ on the frequency of the sojourn time interval in the transitive state. The purpose of FBC is to divide the sojourn time intervals into two groups: normal state with a certain frequency value and abnormal state with a frequency value equal to 0. Increasing δ enlarges the width of neighborhood. Therefore, more and more data influence the frequency, and the new frequency of each sojourn time interval increases. We vary δ from 1 to 100. The result is displayed in Figure 10. When δ is equal to 1 or 3, the sojourn time intervals are divided into two consecutive groups. One group contains the sojourn time intervals from 0 to 299 seconds, for which the frequencies of the intervals are larger than 0. The other is from 300 to over 500 seconds, for which the frequencies of the intervals are equal to 0. When δ is equal to 10 or 100, the sojourn time intervals are divided into more than three groups with consecutive time intervals. Therefore, we select 3 and 300 seconds as the best δ and STO, respectively.

**Figure 11.**
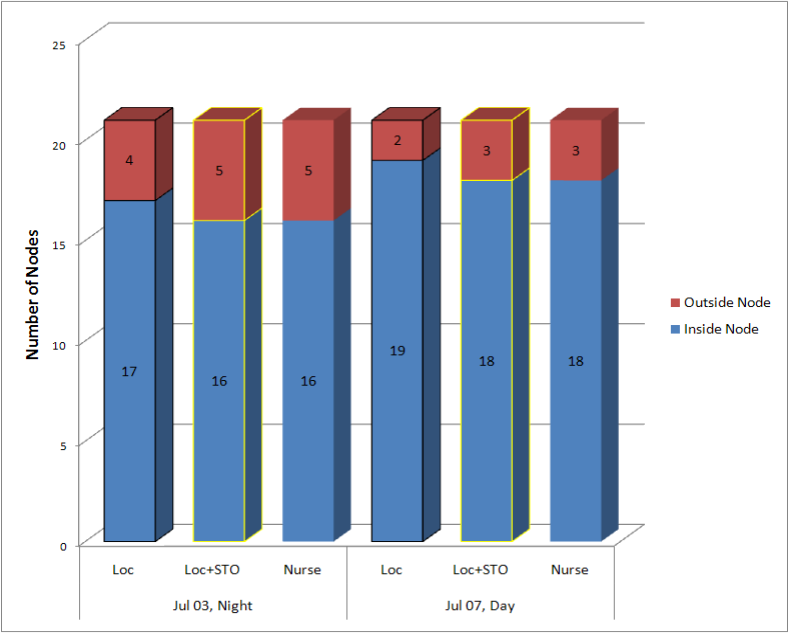
Failure detection of inside node.

**Failure detection of inside node**: We compare the number of inside nodes computed using both STO and the locations of mobile nodes with that of inside nodes computed using the locations of the nodes. Figure 11 shows the comparison results. We set STO to five minutes, as extracted from Figure 10. “Loc” means that the application uses the location of the mobile node to determine node status. “Loc + STO” means that the application uses both the location and the sojourn timeout in the transitive state of the mobile node to determine node status. “Nurse” indicates the number of nodes counted by the nurses. The night and day represent the time shift of the nurses.

The first bar indicates that 17 out of 21 nodes are inside the monitoring area and four nodes are outside the area when the night group checked the number of mobile assets on July 3 with the mobile asset tracking system. However, the state classifier determines that one node has an abnormal transitive state and changes its status from inside to outside when we apply our method to the same date and group. Therefore, the second bar on that date shows that 16 out of 21 nodes are inside and five nodes are outside. The third bar generated by the nurses on that date confirms that our method detects and corrects all incorrect statuses that the system produces by using only the location data. The result of July 7 is similar to that of July 3.

### 5.3. Status Change from Outside to Inside

In this section, we evaluate the impact of the battery lifetime of a mobile node. The battery lifetime is used for separating normal and abnormal nodes. If the estimated battery life time is larger than a certain threshold, the estimator determines that the node has an exhausted battery, and changes node status from outside to inside. The battery life time estimator is useful to detect an abnormal node that stays outside for a long time without battery change. We used a lithium battery, CR123A, made by Panasonic. The battery and node properties related to the current consumption are displayed in Table 4.

**Figure 12.**
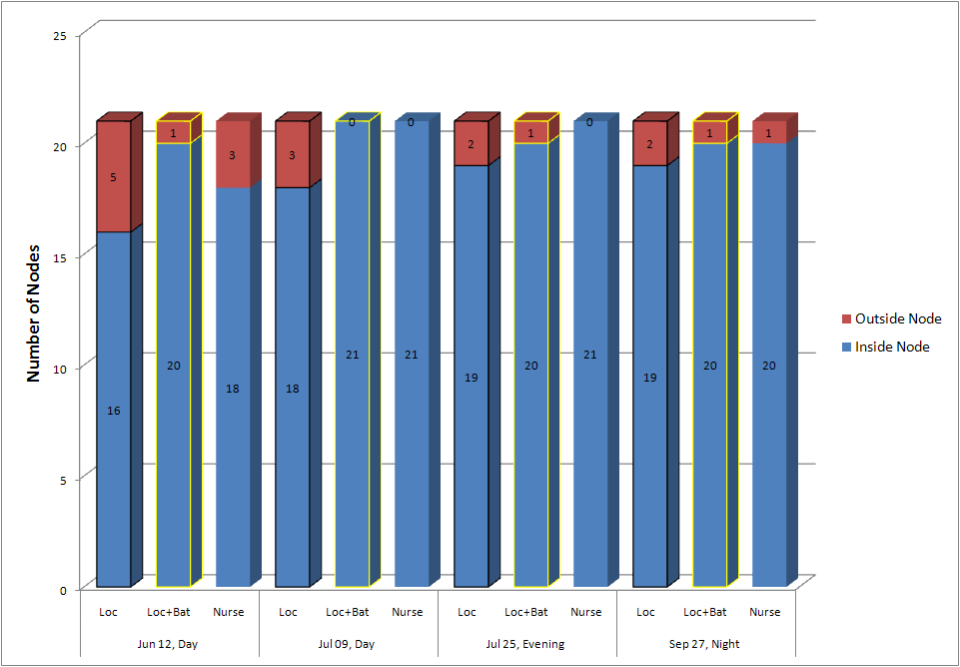
Failure detection of outside node.

We compare the number of outside nodes computed using both the battery lifetime and the location of a mobile node with that of outside nodes based on only the location of one node. Figure 12 shows the comparison results. “Loc + Bat” means that the application uses both the location and the battery life time of the node to determine its status.

The first bar means that three out of 21 nodes are outside the monitoring area and 18 nodes are inside when the day group checked the number of mobile assets on July 9 with the mobile asset tracking system. However, the battery lifetime estimator determines that three nodes have exhausted batteries and changes their status from outside to inside when we apply our method to the same date and group. Therefore, the second bar on that date shows that no node is outside. The third bar generated by the nurses on that date confirms that our method detects and corrects all incorrect statuses that the system produces by using only the location data. The result of September 27 is similar to that of July 9.

The results of June 12 and July 25 show the incorrect determination about the statuses of mobile assets. When the day group checked the number of mobile assets on June 12 with the system, the first bar shows that five nodes are outside the monitoring area and 16 nodes are inside. The second bar on that date shows that only one node is outside because our method changes the four outside nodes’ statuses from outside to inside. However, the third bar generated by the nurses on the date shows that three nodes are outside and 18 nodes are inside. Our method determines the statuses of two nodes incorrectly. We consider the two nodes to have halted. A similar pattern may be observed on July 25. However, it is difficult to classify a disappeared mobile node from the sensor network as either a left node from the monitoring area or a halted node within the area because the gateway reports the same connection state on them. Therefore, we need additional work in the future to solve the problem.

## 6. Conclusions

In this paper, we propose methods to detect and correct incorrect in/out statuses of mobile nodes attached to mobile assets. If a mobile asset tracking system uses the position of a mobile node without being aware of possible incorrect in/out status, the system can generate errors. We tackle this problem to produce more reliable results.

We propose two state classifiers, network-based and frequency-based classifiers, which are applied to the in status. They categorize a transitive state as either normal or abnormal. Each classifier determines a sojourn timeout to discriminate between these two states. The network-based classifier uses certain properties of the sensor network: local waiting time between a parent and a mobile node, and entire waiting time among gateway and reference nodes. The frequency-based classifier uses the number of nodes that stay in the transitive state during a specific period.

We design a battery life time estimator that is applied to the out status. The estimator categorizes a mobile node as inside or outside by using a battery timeout. The battery timeout is computed by the number and duration of stays inside and outside and by the amount of the current consumption of each type of status.

We compare our schemes to a conventional method that uses only the location data to determine in/out statuses of mobile nodes. Experimental evaluation on real data sets shows that our approach recognizes several nodes that are determined to be in incorrect statuses and that it then corrects their statuses. By recognizing and correcting the incorrect in/out statuses, we can improve the in/out determination process for the statuses of mobile assets. We also increase the reliability of the mobile asset tracking application and thereby improve customer satisfaction.

In addition, although this study uses 29 reference nodes and 21 mobile nodes, the system can be easily expanded to monitor increased assets within an enlarged area. As the number of nodes that a gateway can have is limited due to node density, address space, and data collision in the air, we can divide the monitoring area into several sub-areas or into different floors and install one gateway in each sub-area to manage the increased assets. Then, the application gathers data from each gateway and the proposed method is applied to the gathered data. Future work will advance the proposed method to classify a disappeared mobile node from the sensor network as either a left node from the monitoring area or a halted node as well as investigate how well the method works in the case.

## Figures and Tables

**Table 1.** Incorrect in/out status.

**Real Location**	**Application’s Decision**	**Reason**

Outside	Inside	State transition error Protocol error Faulty H/W or S/W
Inside	Outside	Exhausted battery Faulty H/W or S/W

**Table 2.** List of variables.

**Symbol**	**Description**

C_s_,C′_s_	amount of consumed current in sleeping phase
C_p_,C′_p_	amount of consumed current in polling phase
C_t_	amount of consumed current in transmitting phase
T_s_,T′_s_	duration of sleeping phase
T_p_,T′_p_	duration of polling phase
T_t_	duration of transmitting phase
T_c_,T′_c_	duration of one cycle
C_b_	initial battery current

**Table 3.** Component and legend of the mobile asset tracking system.

**Component**	**Count**	**Icon/Figure**	**Meaning**	**Section**	**Patient Types**
Application	1	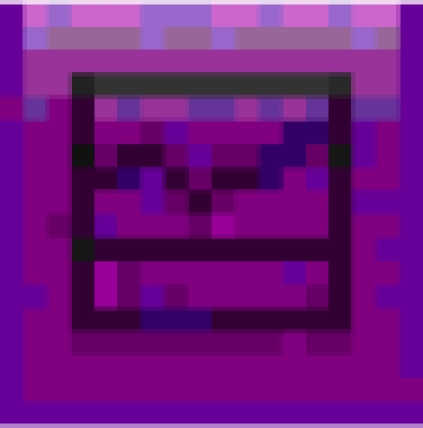	Ventilator	Red	Unconscious or Injured
Gateway	1	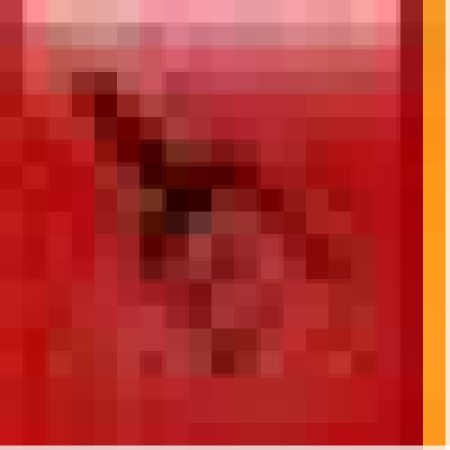	Syringe Pump	Green	Children
Reference node	29	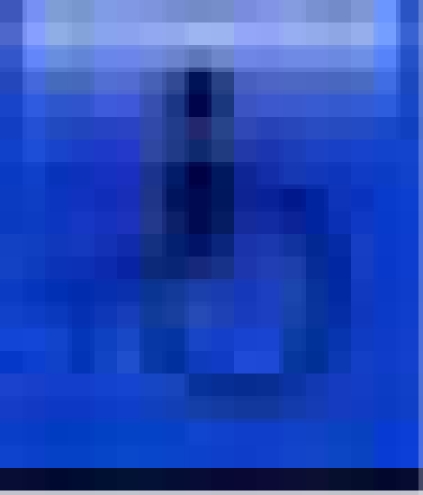	Wheelchair	Orange	Elderly
Mobile node	21	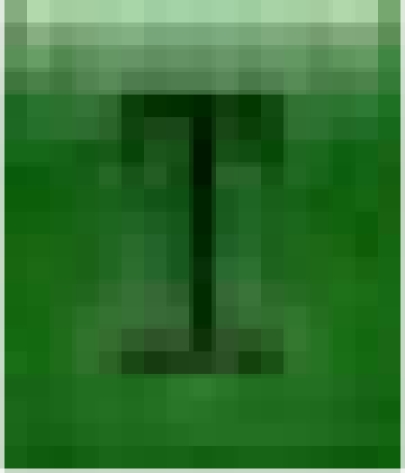	IV Pole	Yellow	General
		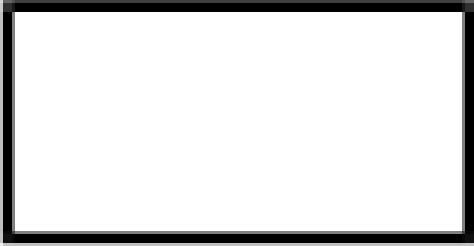	Bed		
		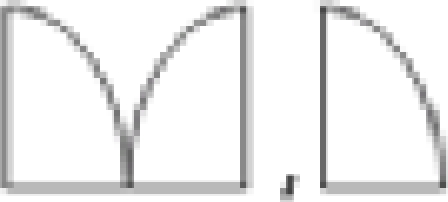	Door		

**Table 4.** Summary of battery and node properties.

**Parameter**	**Value**	**Parameter**	**Value**

C_b_	1300 mAh	b_e_	0.8
C_s_	0.07 mA	C′_s_	0.07 mA
C_p_	12.5 mA	C′_p_	12.5 mA
C_t_	40 mA	T_t_	2 sec
T_s_	30 sec	T′_s_	20 sec
T_p_	5 sec	T′_p_	0.5 sec
C_avg_	3.908 mA	C′_avg_	0.373 mA

## References

[1] Akyildiz I.F., Su W., Sankarasubramaniam Y., Cayirci E. (2002). A Survey on Sensor Networks. IEEE Commun. Mag.

[2] Chintalapudi K., Fu T., Paek J., Kothari N., Rangwala S., Caffrey J., Govindan R., Johnson E. (2006). Monitoring Civil Structures with a Wireless Sensor Network. IEEE Internet Comput.

[3] Sazonov E., Janoyan K., Jha R. Wireless Intelligent Sensor Network for Autonomous Structural Health Monitoring.

[4] Ituen I., Sohn G. (2007). The Environmental Applications of Wireless Sensor Networks. Inter. J. Content.

[5] Mainwaring A., Polastre J., Szewczyk R., Culler D., Anderson J. Wireless Sensor Networks for Habitat Monitoring.

[6] Osterlind F., Pramsten E., Roberthson D., Eriksson J., Finne N., Voigt T. Integrating Building Automation Systems and Wireless Sensor Networks.

[7] Rajendran N., Kamal P., Nayak D., Rabara S. A. WATS-SN: A Wireless Asset Tracking System using Sensor Networks.

[8] Shuai M., Xie K., Ma K., Song G. An On-Road Wireless Sensor Network Approach for Urban Traffic State Monitoring.

[9] Zhuang L.Q., Liu W., Zhang D.H., Kamajaya I. Distributed Asset Tracking using Wireless Sensor Network.

[10] Kim K., Jun J., Kim S., Sung B. Y. Medical Asset Tracking Application with Wireless Sensor Networks.

[11] Mason A., Al-Shamma’a A. I., Shaw A., Irven J., Wiktorowicz R. (2007). Intelligent Wireless Asset Tracking of Packaged Gases. J. Phys.: Conf. Ser.

[12] Klingbeil L., Wark T. A Wireless Sensor Network for Real-time Indoor Localisation and Motion Monitoring.

[13] Yuce M. R., Ng P. C., Lee C. K., Khan J. Y., Liu W. A Wireless Medical Monitoring Over A Heterogeneous Sensor Network.

[14] Chen J.R., Kher S., Somani A. Distributed Fault Detection of Wireless Sensor Networks.

[15] Ramanathan N., Chang K., Kapur R., Girod L., Kohler E., Estrin D. Sympathy for the Sensor Network Debugger.

[16] Rost S., Balakrishnan H. Memento: A Health Monitoring System for Wireless Sensor Networks.

[17] Meier A., Motani M., Siquan H., Kunzli S. DiMo: Distributed Node Monitoring in Wireless Sensor Networks.

